# Intranasal Dexmedetomidine as Sedative for Medical Imaging in Young Children: A Systematic Review to Provide a Roadmap for an Evidence-Guided Clinical Protocol

**DOI:** 10.3390/children9091310

**Published:** 2022-08-28

**Authors:** Kato Hermans, Larissa Ramaekers, Jaan Toelen, Koen Vanhonsebrouck, Karel Allegaert

**Affiliations:** 1 Department of Public Health and Primary Care, Academic Centre for Nursing and Midwifery, KU Levuen, 3000 Leuven, Belgium; 2 Child and Youth Institute, KU Leuven, Herestraat 49, 3000 Leuven, Belgium; 3 Department of Development and Regeneration, KU Leuven, 3000 Leuven, Belgium; 4 Department of Pediatrics, University Hospitals UZ Leuven, 3000 Leuven, Belgium; 5 Department of Pharmaceutical and Pharmacological Sciences, KU Leuven, 3000 Leuven, Belgium; 6 Department of Hospital Pharmacy, Erasmus MC, 3000 GA Rotterdam, The Netherlands

**Keywords:** procedural sedation, children, dexmedetomidine, imaging

## Abstract

There is an increasing need for effective anxiety and pain reduction during medical imaging procedures in children, addressed by non-pharmacological or pharmacological approaches. Dexmedetomidine is a fairly recently marketed, selective α2-adrenergic agonist that can be administered intranasally. To develop an evidence-guided clinical protocol, we investigated the (side) effects, preconditions and safety aspects following intranasal dexmedetomidine administration in children (1 month–5 years) for procedural sedation during medical imaging. To this end, a systematic search (PubMed, Embase and CINAHL (12/2021)) was performed to identify studies on intranasal dexmedetomidine for procedural sedation for medical imaging (computer tomography and magnetic resonance imaging). Following screening and quality assessment, eight studies were retained. Nasal nebulization was considered the best administration method, dosing varied between 2 and 4 µg/kg (age-dependent) 30–45 min prior to imaging and contraindications or restrictions with respect to oral intake were somewhat consistent across studies. Valid sedation scores and monitoring of vital signs were routinely used to assess sedation and the need for rescue dosing (different approaches), whereas discharge was generally based on Aldrete score (score ≥ 9). Heart rate, blood pressure and saturation were routinely monitored, with commonly observed bradycardia or hypotension (decrease by 20%). Based on these findings, a roadmap for evidence-guided clinical protocol was generated.

## 1. Introduction

The Charter of United Nations on Human Rights clearly states that all measures should be taken to prevent or relieve pain, physical discomfort and emotional distress in children [1]. Anxiety and pain in children are both complex and multidimensional phenomena, as children are confronted with potential separation from their parents or loss of control during hospitalization, in addition to anxiety and pain. The unfamiliar environment and recall of previous experiences may further add to their stress and anxiety. It is therefore important to be aware that more than half of all children who undergo a procedure in the hospital have experienced a prior intense anxiety episode [2,3]. 

The focus on maximal anxiety and pain reduction during an intervention in children was one of the drivers to create the PROSA-team (PROcedural Sedation and Analgesia) within University Hospitals Leuven, as recently described in this journal [4]. During interventions, such dedicated teams apply either non-pharmacological or pharmacological approaches, or both. In addition to distraction, vacuum mattresses or supportive therapy as non-pharmacological interventions, pharmacological interventions include fentanyl, midazolam, and—more recently—dexmedetomidine [4,5]. In particular during imaging procedures, avoiding uncontrolled behavior or movement artifacts is of additional importance.

Dexmedetomidine is a selective α2-adrenoceptor agonist with sedative, anxiolytic, sympatholytic and analgesic-sparing effects [6]. Dexmedetomidine also causes dose-dependent hemodynamic side effects. At lower doses, central effects dominate, leading to a decrease in heart rate and blood pressure. At higher doses, peripheral vasoconstrictive effects predominate, resulting in an increase in systemic vascular resistance and blood pressure, with bradycardia further emphasized [7]. Dexmedetomidine is registered for sedation of adults admitted to intensive care units, whereas its off-label use (‘no recommendation on a posology can be made‘) is becoming more common in children [7,8] due to its perceived ability to result in adequate procedural sedation with a relatively low risk of respiratory depression compared to benzodiazepines or opioids [9]. The minimal influence on respiration combined with the fact that the patient can be easily awakened makes dexmedetomidine an interesting alternative in children [10]. However, its safety and efficacy in infants and children have not yet been formally established [7]. 

Given its emerging use in children, there is a need for indication-specific clinical protocols reflecting the policies related to off-label use of medicines in children [11]. The most common indication for use in children is to facilitate radiological procedures. During such procedures, the child is expected to lie down without movement, as movement can lead to artifacts and poor image quality. Medical imaging can also induce anxiety in the child related to, e.g., noise or environment. Therefore, the aim of the present study was to investigate the (side) effects, preconditions and safety aspects of intranasal dexmedetomidine administration as a sedative for procedural sedation during medical imaging in children from 1 month to 5 years, with the aim of developing a roadmap for an evidence-guided protocol for clinical use. To this end, we conducted a systematic review of intranasal dexmedetomidine use in children in the setting of magnetic resonance imaging (MRI), computed tomography (CT) or nuclear medicine imaging. The study population was limited to children between 1 month and 5 years of age, as newborns likely have a specific risk profile, whereas children over 5 years of age are, in general, already more receptive to non-pharmacological interventions. 

## 2. Materials and Methods

### 2.1. Selection Criteria and Systematic Search Strategy

We included studies of any type of design written in English, French or Dutch and published within the last decade. Study selection criteria were (1) children from one month to five years of age, irrespective of comorbidities; (2) use of intranasal dexmedetomidine for procedural sedation during imaging (MRI, CT and nuclear medicine imaging); and (3) occurring in pediatric departments in hospitals, pediatric day care hospitals or radiology centers. We excluded studies in which intranasal dexmedetomidine was administered to neonates and preterm infants, as well as studies in which there an additional intervention was applied during medical imaging (such as CT-guided puncture). The literature review was conducted in December 2021. Pubmed, Embase and CINAHL were consulted for to identify studies on dexmedetomidine use in children. To establish a search string, five main concepts were included:

1. Child, up to the age of five years;
2. Procedural sedation;
3. Dexmedetomidine;
4. Intranasal administration; and
5. Medical imaging.

Index terms (MeSH, Emtree and Subject Heading) were added to each concept, as well as free text words and synonyms. Terms and words were interrelated using the Boolean term ‘OR’. Examples of index terms that were used are: ‘Child, preschool’, ‘Conscious sedation’, ‘Hypnotics and Sedatives’, ‘Nasal absorption’ and ‘Diagnostic imaging’. Free text words and synonyms, such as ‘Infant’, ‘Moderate Sedation’, ‘alpha 2 Adrenergic Receptor’, ‘Intranasal administration’ and ‘Radiologic and Imaging Nursing’, were added. Finally, these five concepts were combined in one search string using the Boolean term ‘AND’. In addition to this search string, we also applied the snowball method, as references of studies that were retrieved were also verified. In addition, we were assisted by an expert, who also provide us with relevant publications. 

### 2.2. Screening Process

After the studies were identified and duplicates were removed, two researchers (K.H. and L.R.) individually reviewed all search results. Disagreements were discussed first, and if inconclusive, the decision was left to a third reviewer (K.A.). The first screening phase consisted of including studies based on title and abstract. Each researcher screened the studies individually and labeled them with ‘0′ (not relevant) or ‘1′ (possibly relevant). Articles labeled with ‘1′ were carried forward to the next screening phase. The second phase involved the inclusion of studies based on full-text assessment. Again, a score of ‘0′ was used for exclusion, and a score of ‘1′ was used for inclusion. 

### 2.3. Quality Assessment and Data Extraction

The next step in the process was to assess the quality and, subsequently, extract all relevant information related to the research questions of the included articles. Quality assessment was based on standardized tools related to the study design. The Jadad scale was used for randomized controlled trials (RCTs) [12], the Newcastle–Ottawa scale (NOS) for nonrandomised studies [13], the National Institute of Health (NIH) quality assessment tool for observational cohort and cross-sectional studies [14] and the AMSTAR-2 score for systematic reviews [15]. Extraction was structured by means of a data extraction tool. With this tool, data were collected for each study, namely title, author and year; study design and characteristics; sample and inclusion/exclusion criteria; setting and type of medical imaging; administration method, time and dose; assessment tools; possible side effects; and results. Articles were analyzed individually by both researchers and combined in the data extraction tool to facilitate comparison between studies. We subsequently applied the PRISMA approach [16]. 

## 3. Results

### 3.1. Selection Criteria and Systematic Search Strategy

**Figure 1:**
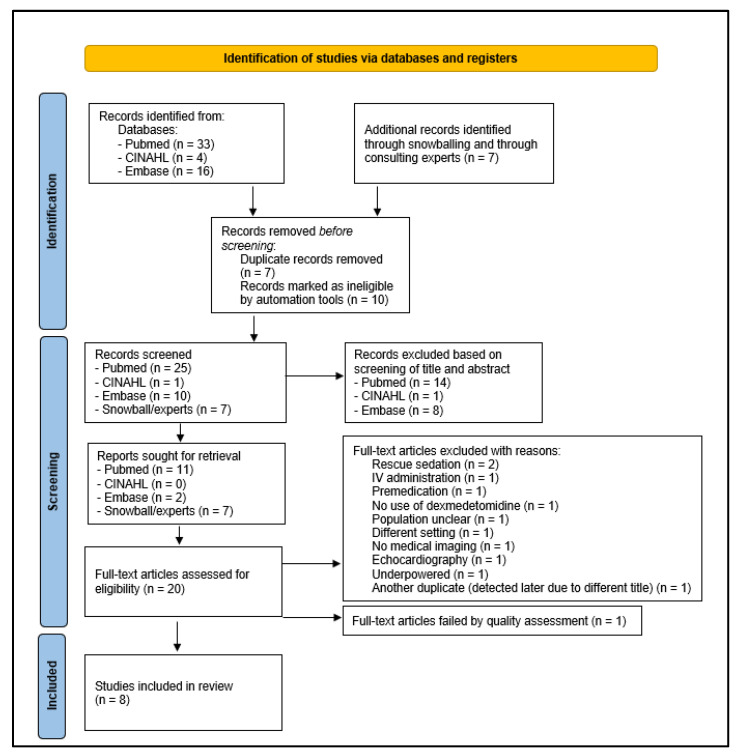
PRISMA 2020 flow diagram [16].

The results of our search strategy are summarized in a PRISMA flow diagram (Figure 1). Most studies were retrieved from PubMed, although some additional studies were found via the snowball method or were provided by an expert (K.A.). We identified 60 studies in total. Of these, seven duplicates were removed, as well as ten studies that did not meet predefined filters for language and publication date (automation tools). In the first screening phase, 23 studies were excluded (title–abstract screening), and 11 were dropped during the second phase (full-text screening). Ultimately, one study failed upon quality assessment (3.2.), so eight studies (2012–2021) were retained for data extraction (Figure 1).

### 3.2. Quality Assessment

Nine studies were retained after screening and underwent quality assessment with an assessment tool adapted to their respective study design. Three RCTs were assessed using the Jadad scale (score 0–5; a score < 3 reflects insufficient reporting on methodological quality) (Supplemental Materials, Table S1). All assessed RCTs scored well in terms of methodological quality and were retained for data extraction, with a score of 5/5 for two RCTs [12,17,18], or 4/5 (blinding methodology not sufficiently well described) [12,19]. One study was analyzed based on the NOS scale (eight items related to ‘selection’, ‘comparability’ or ‘outcome’). This effort resulted in a score of seven stars, reflecting good methodological quality (Supplemental Materials, Table S2) [13,20]. Four studies were evaluated using the ‘NIH Quality Assessment Tool for Observational Cohort and Cross-Sectional Studies’ (Supplemental Materials, Table S3) [14,21,22,23,24]. This tool provides a list of questions to reflect on key concepts of internal study validity. Although some items were not always reported, could not be determined or were not applicable, all studies achieved a score of at least eight. Finally, one systematic review was assessed using the ‘AMSTAR 2 Checklist’ (Supplemental Materials, Table S4) [15,25]. This scale aims to provide an overview of weaknesses within critical areas, as well as potential bias elements. With all assessors involved (K.H., L.R. and K.A.), we concluded that this publication insufficiently reported on their methodological approach in 10/16 items. We therefore decided not to retain this paper, so eight papers ultimately underwent data extraction.

### 3.3. Data Extraction 

A complete overview of all characteristics of the individual studies (purpose and outcomes, study design and characteristics, type of imaging and sample size, inclusion and exclusion criteria, administration method, timing and dose and outcome variables, including assessment tools, is provided in the data extraction tool (Supplemental Materials, Table S5).

#### 3.3.1. Purpose of Studies and Outcomes

Four studies explored the effectiveness of dexmedetomidine, representing the most commonly studied aspect. However, there were some differences in indications in terms of which type of dexmedetomidine was administered (premedication to facilitate preparatory interventions, such as transfer; intravenous infusion; or primary sedation for medical imaging). The focus of some studies was somewhat broader, such as safety aspects or recovery time. 

One study sought to determine the effectiveness of intranasal dexmedetomidine administration as the sole premedication prior to a CT scan [17]. Another study evaluated the efficacy (sedation and imaging quality), safety (hemodynamics and saturation) and outcome (discharge) of intranasal dexmedetomidine administration for CT scanning [22]. Ambi et al. examined the efficacy (University of Michigan Sedation Scale (UMSS) ≥2 to enable transfer and child–parent separation) of a specific intranasal dose (2 µg/kg) of dexmedetomidine for MRI [21]. Tug et al. explored the dose–response pattern, assessing the Ramsay sedation score (RSS), hemodynamics, saturation and respiratory rate (need for propofol rescue and recovery) following intranasal administration of either 3 or 4 µg/kg dexmedetomidine [18]. Yuen et al. compared oral (50 mg/kg) chloral hydrate administration to intranasal (3 µg/kg) dexmedetomidine administration 30 min before CT imaging on the bases of sedation (UMSS), discharge criteria (Aldrete score) and tolerance (palatability and vomiting). An audit study described the use of both intranasal dexmedetomidine (2.5–3 µg/kg) and midazolam (0.29–0.39 mg/kg) for MRI sedation [24]. Similarly, Jackson et al. audited their outcome (imaging quality) of MRI sedation in three consecutive protocols [20]. In one of these, intranasal dexmedetomidine was used as the sole sedative. This section was included in the systematic review. Finally, Uusalo focused on intranasal dexmedetomidine (2–3 µg/kg) pharmacokinetics and pharmacodynamics (comfort-B score and vital signs) [23].

Primary outcomes were time to recovery after sedation [19], adequate sedation (RSS ≥ 4, no need for rescue intravenous sedation) [17], mean time until sedation [18], successful sedation (imaging quality) [20], response to child–parent separation [21], efficacy measures (number of doses, time to achieve sedation and time to meet discharge criteria) [22], major adverse events [24] and pharmacokinetics [23]. In addition to sedation and discharge scores, secondary outcomes related to safety (hemodynamics and respiratory rate), tolerance (palatability and vomiting), imaging quality, need for additional dosing and parental outcomes were considered. 

#### 3.3.2. Study Design and Characteristics, Sample Size, and Inclusion and Exclusion Criteria

Study designs included RCTs and prospective or observational studies/audits. Three of these were double-blinded and two were single-blinded, whereas three other studies were observational and not blinded. Five studies were prospective, and four were retrospective, such as the audit of Jackson, reporting both retrospective and prospective data. Finally, one non-randomized, ‘open-label’ exploratory study was included. 

Five studies were related to MRI procedures, and three involved CT scans. We observed a considerable variability in terms of sample size (range 28–256) between the studies. The age category of included studies was mostly on target (1 month–5 years), with minor deviations with increased age. Inclusion criteria between studies were rather consistent. For example, American Society of Anesthesiologists (ASA) status I/II, scheduled imaging, informed consent and patient age were commonly mentioned, with additional study-specific elements (peripheral catheter access, language skills or operator). With respect to exclusion criteria, we noticed more differences across studies. The following exclusion criteria were commonly present: allergy to study drugs or other products used; history of cardiac dysfunction or respiratory problems; presence of ear, nose and Throat (ENT) diseases; severe organ dysfunction or chronic drug use, along with study-specific elements (previous administration of sedatives, risk of vomiting or aspiration and presence of reflux). 

#### 3.3.3. Method of Administration, Timing and Dose

Two options for intranasal dexmedetomidine administration were reported. A mucosal nebulizer/nose spray was used in four studies, with instillation into the nostril using a tuberculin syringe or similar device reported in the other studies. Both Tug et al. and Ambi et al. stated that nebulizing is more effective (covers a larger area, with higher bioavailability) [18,21]. There was some variability in the time of administration to planned imaging, from 30 to 60 min. Four studies implemented a pre-procedure nothing per os (NPO) policy. There was some difference in terms of the hours of solid and artificial feeding between studies. For liquids, there was more consistency (2 h). The dose used within the studies ranged from 2 to 4 μg/kg. The lowest dose (2 μg/kg) was used by Ambi et al. [21]. Jackson et al. distinguished between <15 kg and ≥15 kg (2 and 4 μg/kg) [20]. Ghai et al. and Filho et al. used 2.5 μg/kg [17,22]. If the RSS was still too low (<3), a second dose (1 μg/kg) was administered [22]. Another study used different doses for different age categories, with an average dose of 2.7 μg/kg for the 0–2 years group and 2.9 μg/kg in the 2–6 years group [23]. Yuen et al., Sulton et al. and Tug et al. used a dose of 3 μg/kg in an RCT design compared to a dose of 4 μg/kg [18,19,24]. 

#### 3.3.4. Outcome Variables

Several physiological parameters (oxygen saturation, heart rate, blood pressure and respiratory rate) were repeatedly or continuously measured. These parameters were commonly collected as safety markers. Only Filho et al. reported hypoxia in one patient [22]. In contrast, a decrease in heart rate was more common, up to 20% lower compared to usual or initial values. Related to blood pressure, a decrease reflecting hypotension was common (9% and 10% in the Filho et al. and Yuen et al. studies, respectively), although a decrease > 20% was very rare (one case in the Filho et al. study) [19,22]. The most commonly used assessment tools to quantify the degree of sedation were the UMSS and RSS tools. Threshold values for top-up dosing showed some variability. Other tools applied were the bispectral index score (BIS monitor) and the comfort-B score. The Aldrete score was commonly applied to determine when a patient was fit for discharge (minimum score: 9). Other outcome variables were related to imaging quality or focused on parental outcomes (Parental Separation Score, Parental Satisfaction Score). 

## 4. Discussion

Studies on intranasal dexmedetomidine as a sedative for medical imaging in young children were only retrieved for MRI and CT procedures in this review. However, we assume that we can extrapolate these findings to nuclear medicine imaging. Both MRI and CT are associated with various individual levels of anxiety due to separation, unfamiliar environment or recall. The duration is commonly much longer for MRI procedures, and MRI imaging is also associated with significantly more noise than CT scans, which may alter the level and duration of sedation needed to enable the collection of clinically meaningful images. 

A specific aspect of sedation for radiological procedures is that not all children require sedation. Determination of individual patient needs remains a challenge, which is only impartially reflected by age. Assessing individual capabilities and needs and matching them to what is needed for a successful procedure is a skill [26]. Related to this, we predefined a subgroup (1 month–5 years) as priority, assuming that this subgroup commonly requires pharmacological sedation, despite the existence of non-pharmacological tools. This does not mean that these non-pharmacological tools are not valuable in this age category, as they can either reduce or even replace drug exposure, with the promise of improving self-esteem and self-control in children. Procedure-specific tools, include MRI mockup scanners, video or virtual reality systems, ear protection to distract and reduce stress in patients and the use of a vacuum mattress, among others [2,3,4,26].

Intranasal dexmedetomidine dosing ranged between 2 to 4 µg/kg, without a clear difference between imaging modalities (CT: 2.5–3 µg/kg [17,19,22]; MRI: 2–4 µg/kg [18,20,21,23,24]), despite the clinical rationale to discriminate between the two procedures [20,24], whereas Jackson used a weight-based (15 kg threshold) in applied dosing strategy. An assessment of all retrieved information suggests that dose decisions for clinical protocol development depend, in part, on the level and type of efficacy/safety targeted outcome variables (effective sedation versus time to discharge and side effects). A lower dose (2 µg/kg, MRI) resulted in no side effects, with additional dosing in 40% of patients [21]. Along the same line, a 2.5 µg/kg dose (CT) resulted in the necessity of additional dosing in 33% of patients [17]. Higher doses (3–4 µg/kg) were more effective when the need for additional dosing was used as an outcome [18] but would likely result in a somewhat prolonged time until return to normal activities (4.3 h) [19], as Tug did not document a difference in recovery time (Aldrete score) between the 3 and 4 µg/kg groups [18]. 

Besides the twofold range in dosing, the literature is not fully conclusive on the administration method of intranasal dexmedetomidine. The use of nebulization or instillation was divided proportionally among the reviewed studies. Two studies that opted for instillation mentioned in their discussion that nebulization is the preferable option because it is reasonable to assume that nebulized particles cover a larger surface area compared to nasal instillation, resulting in improved bioavailability [18,21]. A pharmacokinetic study used a nebulizing medical device [23]. The time of administration varied across studies, between 30 and 60 min before initiation of the imaging procedure. 

Besides dose selection and type and timing of administration, some ‘circumstantial’ and ‘procedural’ aspects were also considered. Circumstantially, it is common to apply an NPO policy. All reviewed studies prohibited clear liquids two hours before the procedure but with less uniformity with respect to milk feeling and more diversity in terms of limits for solid foods. Procedural practices relate to sedation and recovery assessment, monitoring and exclusion criteria. To explore the sedation level, various measuring tools were used. RSS was applied in four studies, with different thresholds, i.e., higher for MRI than for CT (RSS of ≥4 or ≥5 for MRI [18,20]; RSS ≥3 or ≥4 for CT [17,22]. The (modified) Aldrete (score ≥9) was most commonly used metric to assess recovery. In the majority of studies, physiological parameters were monitored before and after dexmedetomidine administration, with diversity in time intervals at which parameters were collected. Continuous monitoring of heart rate, blood pressure and oxygen saturation was commonly applied. Vomiting was an adverse event of special interest. This is perhaps because vomiting has been reported as a common adverse event following chloral hydrate administration. Alternatively, vomiting a relevant patient outcome variable. Exclusion criteria related to drug allergies, ASA status III/IV and hepatic abnormalities. In addition, patients with cardiac abnormalities, central nervous system dysfunction, respiratory and renal dysfunction, mental retardation or a risk of difficult intubation (ENT) are sometimes excluded from clinical studies.

Systematic reviews have strengths but are obviously also subject to limitations. First, statistically significant findings retrieved in this systematic review are different in terms of clinical relevance when considered for clinical protocol development. For example, a 2.8 min difference between dexmedetomidine to chloral hydrate in time to sedation was statistically significant but is not of clinical relevance [19]. In contrast, adequate sedation or successful imaging [17,20,24], parental separation distress [18] or palatibility aspects [19] are more (patient) relevant outcome variables. Secondly, we were not always able to extract the data specific for the age category we predefined, specifically for the higher age range. We attempted to address this by sending e-mails to the corresponding authors. As did not receive any response, we decided to focus on the mean or median age of the cohorts. Finally, our search strategy yielded only 60 results, possibly due to a narrow search string or the fact that the medicine under investigation was only recently introduced for pediatric procedural sedation. Furthermore, the ratio of articles from databases to those recommended by an expert was somewhat imbalanced. 

Considering these limitations and based on the outcome of the systematic review, we are confident to conclude that the roadmap suggested to develop an evidence-guided clincial protocol for intranasal dexmedetomidine use in young children is robust, as it is based on a systematic review of the available literature on this topic (Table 1). 

## Figures and Tables

**Table 1:** A suggested roadmap with respect to aspects to consider in the development of an evidence-guided clinical protocol on intranasal dexmedetomidine use in young children.

Aspect	Suggestions	
**Contraindications**	ASA-status III/IV; hepatic abnormalities; cardiac abnormalities; central nervous system dysfunction; respiratory or renal dysfunction; risk of difficult intubation; drug allergies	
**Dose**	Age-related suggestion: 2,5 µg/kg under 1 year, 3 µg/kg in 1 to 3 years, 4 µg/kg in 3 to 5 years Modality-related suggestion: CT: 2.5–3 µg/kg; MRI 2–4 µg/kg	
**Administration method**	Nebulization by mucosal spray	
**Timing of administration**	30–45 min before the procedure	
**NPO policy**	Variability in practices - Prohibition of clear liquid consumption one or two hours before sedation/procedure - Prohibition of milk food consumption four to six hours before sedation/procedure - Prohibition of solid food consumption eight hours before sedation/procedure In case of an emergency, consider specific guidelines, such as the American College of Emergency Physicians guidelines.	
**Sedation monitoring**	A valid sedation scale should be used. Consider the use of the Ramsay sedation score (RSS) or the University of Michigan Sedation Scale 10 and 20 min after administration, respectively. For the RSS score and to obtain adequate sedation, this score should be as follows: for MRI: ≥4 or ≥5; for CT: ≥3 or ≥4.	
**Rescue medication**	Several options were suggested in the reviewed studies, including (1) an additional dose of dexmedetomidine, (2) intravenous bolus propofol or thiopental or (3) intravenous/intranasal midazolam. Owing to this inconsistency, we cannot provide further guidance on selection of rescue medication.	
**Discharge**	Modified Aldrete score has to be registered after the procedure (threshold score ≥9) for discharge.	
**Monitoring**	Continuous monitoring of heart rate and oxygen saturation is highly recommended throughout the procedure. If an abnormal heart rate is observed, blood pressure should also be measured. Blood pressure is not measured by default, as it can result in arousal and is a late indicator of circulatory failure.	
**Potential side effects**	Commonly occurring	Bradycardia, with a decrease of <20%
		Hypotension, with a decrease of <20%
	Less commonly occurring	Bradycardia, with a decrease of >20%
		Hypotension, with a decrease of >20%
		Vomiting
	Rarely occurring	Desaturation, hypertension and tachycardia
**Non-pharmacological** **interventions**	Consider simultaneous use of non-pharmacological interventions, such as hearing protection, use of a vacuum matress or a distraction or mockup	

## Data Availability

Not applicable.

## Supplementary Materials

The following supporting information can be downloaded at: https://www.mdpi.com/article/10.3390/children9091310/s1, Table S1: Overview assessments on the Jadad scale, Table S2: Overview assessment on the Newcastle–Ottawa scale, Table S3: Overview assessments based on the National Institute of Health (NIH) Quality Assessment Tool, Table S4: Overview assessment based on AMSTAR 2, Table S5: Data extraction of the eight studies retained in the systematic review, reporting on purpose and outcomes, study design and characteristics, type of imaging and sample size, inclusion and exclusion criteria, administration method, timing and dose and outcome variables, including assessment tools.
