# Supplementary material for: Intranasal Dexmedetomidine as Sedative for Medical Imaging in Young Children: A Systematic Review to Provide a Roadmap for an Evidence-Guided Clinical Protocol
Source: Children (Basel). 2022 Aug 28;9(9):1310. doi: 10.3390/children9091310 (PMC9498011; doi:10.3390/children9091310)
Supplement: Supplementary file 1 [file children-09-01310-s001.zip › children-1894539-supplementary.pdf]

Supplemental Materials

Table S1. Overview assessments Jadad Scale [12,17-19]

Jadad Scale			
**Questions**	**Yuen et al. 2017 [19]**	**Ghai et al. 2016 [17]**	**Tug et al. 2015 [18]**
Was the study described as randomized (this includes words such as randomly, random, and randomization)?	Yes	Yes	Yes
Was the method used to generate the sequence of randomization described and appropriate (like table of random numbers, computer-generated)?	Yes	Yes	Yes
Was the study described as double blind?	Yes	Yes	Yes
Was the method of double blinding described and appropriate (like identical placebo, active placebo, dummy)?	Not described	Yes	Yes
Was there a description of withdrawals and dropouts?	Yes	Yes	Yes
**FINAL SCORE**	**4**	**5**	**5**

Table S2. Overview assessment Newcastle-Ottawa Scale (NOS) [13,20]

NOS	
**Items**	**Jackson et al. 2021 [20]**
**Selection**	
- Representativeness of the exposed cohort	☆
- Selection of the non-exposed cohort	
- Ascertainment of exposure	☆
- Demonstration that outcome of interest was not present at start of study	☆
**Comparability**	
- Comparability of cohorts on the basis of the design or analysis	☆☆
**Outcome**	
- Assessment of outcome	☆
- Was follow-up long enough for outcomes to occur	☆
- Adequacy of follow up of cohorts	
**FINAL SCORE**	**GOOD QUALITY**

Table S3. Overview assessments National Institute of Health (NIH) Quality Assessment Tool [14,21-24]

**NIH Quality Assessment Tool for Observational Cohort and Cross-Sectional Studies**				
**Questions**	**Ambi et al. 2012 [21]**	**Filho et al. 2015 [22]**	**Uusalo et al. 2020 [23]**	**Sulton et al. 2017 [24]**
Was the research question or objective in this paper clearly stated?	Yes	Yes	Yes	Yes
Was the study population clearly specified and defined?	Yes	Yes	Yes	Yes
Was the participation rate of eligible persons at least 50%?	Cannot determine	Cannot determine	Yes	Yes
Were all the subjects selected or recruited from the same or similar populations? Were inclusion and exclusion criteria for being in the study prespecified and applied uniformly to all participants?	Yes	Yes	Yes	Yes
Was a sample size justification, power description, or variance and effect estimates provided?	Not reported	Not reported	Yes	Not reported
For the analyses in this paper, were the exposure(s) of interest measured prior to the outcome(s) being measured?	Yes	Yes	Yes	Yes
Was the timeframe sufficient so that one could reasonably expect to see an association between exposure and outcome if it existed?	Yes	Yes	Yes	Not reported
For exposures that can vary in amount or level, did the study examine different levels of the exposure as related to the outcome?	No	No	Yes	Cannot determine
Were the exposure measures (independent variables) clearly defined, valid, reliable, and implemented consistently across all study participants?	Yes	Yes	Yes	Yes
Was the exposure(s) assessed more than once over time?	Yes	Yes	Yes	Not reported
Were the outcome measures (dependent variables) clearly defined, valid, reliable, and implemented consistently across all study participants?	Yes	Yes	Yes	Yes
Were the outcome assessors blinded to the exposure status of participants?	Not reported	Yes	No	Not applicable
Was loss to follow-up after baseline 20% or less?	Yes	Yes	Yes	Yes
Were key potential confounding variables measured and adjusted statistically for their impact on the relationship between exposure(s) and outcome(s)?	Yes	Not reported	Yes	No
**FINAL SCORE**	10/14 YES	10/14 YES	13/14 YES	8/14 YES

Table S4. Overview assessment AMSTAR 2 [15,25]

AMSTAR 2	
**Questions**	**Lewis et al. 2020 [25]**
Did the research questions and inclusion criteria for the review include the components of PICO?	Yes
Did the report of the review contain an explicit statement that the review methods were established prior to the conduct of the review and did the report justify any significant deviations from the protocol?	Partial yes
Did the review authors explain their selection of the study designs for inclusion in the review?	No
Did the review authors use a comprehensive literature search strategy?	Partial yes
Did the review authors perform study selection in duplicate?	Yes
Did the review authors perform data extraction in duplicate?	No
Did the review authors perform data extraction in duplicate?	No
Did the review authors describe the included studies in adequate detail?	No
Did the review authors use a satisfactory technique for assessing the risk of bias (RoB) in individual studies that were included in the review?	No
Did the review authors report on the sources of funding for the studies included in the review?	No
If meta-analysis was performed did the review authors use appropriate methods for statistical combination of results?	Yes
If meta-analysis was performed, did the review authors assess the potential impact of RoB in individual studies on the results of the meta-analysis or other evidence synthesis?	Yes
Did the review authors account for RoB in individual studies when interpreting/ discussing the results of the review?	Yes
Did the review authors provide a satisfactory explanation for, and discussion of, any heterogeneity observed in the results of the review?	No
If they performed quantitative synthesis did the review authors carry out an adequate investigation of publication bias (small study bias) and discuss its likely impact on the results of the review?	No
Did the review authors report any potential sources of conflict of interest, including any funding they received for conducting the review?	Yes
**FINAL SCORE**	**8 x No**<br>**2 x Partial yes**

Table S5. Data extraction of the eight studies retained in the systematic review, reporting on purpose and outcomes, study design and characteristics, type of imaging and sample size, inclusion and exclusion criteria, administration method, timing and dose, and outcome variables, including assessment tools [17-24]

Title, authors, date	Study design and characteristics	Sample, inclusion and exclusion criteria	Setting, type of imaging	Assessment tools
**A randomised controlled trial of oral chloral hydrate vs. intranasal dexmedetomidine before computerised tomography in children.**<br><br>**Yuen et al. 2017** [19]<br><br>**Purpose of the study**<br><br>To test whether children recovered differently after oral chloral hydrate compared with intranasal dexmedetomidine.	**Design:**<br>Randomised controlled trial<br>**Randomization:**<br>They generated a computerised random sequence to allocate children to:<br>→ PO chloral hydrate<br>+ IN placebo<br>OR<br>→ PO placebo<br>+ IN dexmedetomidine<br>**Blinding:**<br>Pharmacists prepared the study drugs in numbered, indistinguishable containers, the contents of which patients and investigators were blinded to.	**Sample:**<br>n = 196 children<br>→ 87 children received IN dexmedetomidine<br>Average age:<br>32,5 months<br>Average weight:<br>12 kg<br>**Inclusion criteria:**<br>- ASA status I/II<br>- Scheduled CT scan under sedation between March 2013 and February 2015<br>- Informed consent (parents/legal guardian) + child's assent (if mature enough)<br>**Exclusion criteria:**<br>- Allergy to a study drug<br>- History of cardiac arrhythmia, congenital heart disease or severe organ dysfunction	**Setting:**<br>- Queen Mary Hospital in Hong Kong<br>- Guangzhou Woman and Children's Medical Centre in China<br>**Type of imaging:**<br>Computed tomography (CT scan)<br><br>**Method of administration, timing, dose**<br><br>**Method of administration:**<br>Atomised nasal spray (MAD Nasal™ Intranasal Mucosal Atomization Device* - Teleflex)<br>**Timing:**<br>30 minutes before the CT study<br>**Dose:**<br>3 µg/kg intranasal dexmedetomidine	Baseline: response, blood pressure, pulse rate, SpO_2<br>All time recording:<br>- Blood pressure 20% less than normal<br>- Heart rate<br>- SpO_2 < 95% - airway interventions<br>- Episodes of vomiting<br>**University of Michigan Sedation Scale (UMSS)** → *Every five minutes after administration*<br>1 = Awake and alert<br>2 = Sleepy, with appropriate responses to voice<br>3 = Somnolent, roused by touch<br>4 = Asleep, roused by significant stimulation<br>5 = Not rousable<br>**Aldrete score** → *A score of at least 9 = ready for discharge*<br>**- Activity:**<br>2 = Able to move spontaneously/on command 4 extremities<br>1 = Able to move voluntarily/on command 2 extremities<br>0 = Unable to move any extremities<br>**- Respiration:**<br>2 = Able to deep breath and cough freely; 1 = Dyspnea, shallow or limited breathing; 0 = Apneic<br>- **Circulation:**<br>2 = Blood pressure +/- 20% of pre-anesthesia level<br>1 = Blood pressure +/- 20-49% from pre-anesthesia level<br>0 = Blood pressure +/- 50% of pre-anesthesia level<br>**- Consciousness:**<br>2 = Fully awake; 1 = Arousable on calling0 = Not responding<br>**- Skin color:**<br>2 = Normal; 1 = Pale, dusky, blotchy, jaundiced, other; 0 = Cyanotic

<table>
<tr><th>Title, authors, date</th><th>Study design and characteristics</th><th>Sample, inclusion and exclusion criteria</th><th>Setting & type of imaging</th><th>Assessment tools</th></tr>
<tr>
<td>Comparison of oral midazolam with intranasal dexmedetomidine premedication for children undergoing CT imaging: a randomized, double-blind, and controlled study.

Ghai et al. 2016 [17]</td>
<td rowspan="3"><u>Design:</u>
Randomized, double-blind, and controlled study
<u>Randomization:</u>
Allocation to groups using a computer-generated randomization schedule
<u>Blinding:</u>
Randomization schedule was kept in opaque sealed envelopes and opened up by an anesthesiologist not involved in the study, but involved in the clinical management of the children, who prepared the study drug
Investigators were not aware of the group allocation.</td>
<td rowspan="3"><u>Sample</u>
n = 59
→ 30 children received IN dexmedetomidine
<u>Inclusion criteria:</u>
- Children aged between 1 and 6 years
- ASA status I/II
- Scheduled CT scan under sedation
- Written informed consent from parents/legal guardian
<u>Exclusion criteria:</u>
- History of allergy to EMLA cream, midazolam or dexmedetomidine
- Presence of dysfunction of cardiovascular, central nervous, or hepatic system
- Children on chronic hepatic enzyme-inducing drugs
- Mentally retarded children</td>
<td><u>Setting:</u>
Public tertiary care hospital in India
<u>Type of imaging:</u>
Computed tomography (CT scan)</td>
<td rowspan="3"><u>Acceptance of sedation - premedication</u>
- 'Good': easily without resistance
- 'Fair': minor resistance - physical restraint required
- 'Poor': resisting or spitting/vomiting out
<u>Ramsay Sedation Score (RSS)</u>
→ 40 and 50 minutes after EMLA application (= 10 and 20 minutes after administration of premedication)
→ on the CT table
! If not responding to verbal commands, (RSS ≥ 4), nasal prongs were applied and response noted
<u>Monitoring</u>
After administration of premedication:
- SpO_2 and
- NIBP } If no response to nasal prong application
- ECG

<u>Groningen Distress Rating Scale (GDRS)</u>
1 = Calm
2 = Mild distress
3 = Serious distress, in control
4 = Severe distress, out of control
5 = Panic</td>
</tr>
<tr>
<th>Purpose of the study</th>
<th>Method of administration, timing, dose</th>
</tr>
<tr>
<td>To compare the effectiveness of oral midazolam and intranasal dexmedetomidine as <u>sole</u> premedication in children for carrying out both IV cannulation as well as CT scanning.</td>
<td><u>Method of administration:</u>
- Recumbent position
- The drug was dripped into child's nostrils using a tuberculin syringe
<u>Timing:</u>
+/- 30 minutes before the procedure
<u>NPO policy for:</u>
- 8 hours for solid food
- 6 hours for nonhuman milk
- 4 hours for breast feed
- 2 hours for clear fluid
} Prior to the scheduled procedure
<u>Dose:</u>
2,5 µg/kg intranasal dexmedetomidine</td>
</tr>
</table>

Title, authors, date	Study design and characteristics	Sample, inclusion and exclusion criteria	Type of imaging	Assessment tools
**Comparison of Two Different Intranasal Doses of Dexmedetomidine in Children for Magnetic Resonance Imaging Sedation**<br><br>**Tug et al. 2015** [18]<br><br>**Purpose of the study**<br>- To compare two different doses of intranasal dexmedetomidine applied to children for MRI sedation.<br>- To provide effective, well-tolerated sedation for MRI by the intranasal administration of dexmedetomidine.<br>- To reduce propofol requirement and shorten the post-sedation recovery period.	**<u>Design</u>**<br>Prospective, randomized, double-blind study<br>**<u>Randomization:</u>**<br>Random allocation by using a computer-generated table of random numbers.<br>**<u>Blinding:</u>**<br>Study data were collected by two anesthesiology specialists who were blinded to the treatment groups. Parents, the radiology technician and the radiology specialist were also blinded to the treatment groups.	**<u>Sample:</u>**<br>n = 60<br>**<u>Inclusion criteria:</u>**<br>- 1 to 10 years of age; ASA status I/II; Undergoing cranial MRI examinations for various reasons; Informed consent from the patients' parents (written + verbal)<br>**<u>Exclusion criteria:</u>**<br>- Severe cardiac, respiratory, hepatic or renal dysfunction<br>- Risk factors for difficult intubation<br>- Nasal deformity<br>- Hypersensitivity to the medications used<br>- Mental retardation, autism, cerebral palsy, central nervous system disease, active systemic disease, metabolic disorder, or electrolyte imbalance<br>- Severe dehydration or malnutrition<br>- Using analgesics and anticonvulsants during the pre-examination period	**<u>Type of imaging:</u>**<br>Magnetic resonance imaging (MRI scan)<br><br>**Method of administration, timing, dose**<br>**<u>Method of administration:</u>**<br>The drug was dripped into both nostrils using a 1ml syringe<br>**<u>Timing:</u>**<br>45 minutes before the MRI examination<br>**<u>NPO policy:</u>**<br>They were allowed to consume:<br>- Food, including milk, up to 8 hours (for children younger than 36 months of age) or 6 hours (children aged 12-36 months) before.<br>- Clear liquids up to 2 hours before sedation.<br>**<u>Dose:</u>**<br>Group 1: 3 μg/kg<br>Group 2: 4 μg/kg	**<u>Monitoring:</u>** *every 10 minutes*<br>→ heart rate (HR), saturation (SpO_2), respiration rate (RR)<br><br>**<u>Ramsay Sedation Score (RSS):</u>** *10-min intervals*<br>1 = Patient is anxious and agitated or restless<br>2 = Patient is cooperative, oriented, and tranquil<br>3 = Patient responds to commands only<br>4 = Patient exhibits a brisk response to painful stimulus<br>5 = Patient exhibits a sluggish response to painful stimulus<br>6 = Patient exhibits no response<br>→ RSS score ≥ 5 = effective sedation<br>→ RSS score 2 = awakening from sedation<br>**<u>The Bispectral Index score (BIS)</u>**<br>→ At baseline, before and after the procedure and just before discharge<br>= ranging from 0 to 100 (no cerebral activity to fully awake)<br>**<u>Aldrete score:</u>** end of MRI → score 9 = recovery duration<br>**<u>Parental separation score:</u>** *recording of patient's mood*<br>1 = Anxious, irritable<br>2 = Anxious, easily consolable<br>3 = Tranquil/sleepy – sufficient sedation<br>**<u>Parental satisfaction:</u>** *self-reported score*<br>1 = Not satisfied<br>2 = Satisfied<br>3 = Excellent<br>**<u>Three-point scale:</u>** *quality of the MRI examination*<br>1 = No motion<br>2 = Minor movement<br>3 = Major movement (necessitating another scan)

Title, authors, date	Study design and characteristics	Sample, inclusion and exclusion criteria	Setting & type of imaging	Assessment tools
**Dexmedetomidine improves success of paediatric MRI sedation**<br><br>**Jackson et al. 2021** [20]	<u>**Design:**</u><br>Retrospective and prospective study	<u>**Sample:**</u><br>n = 74 children<br>→ 85 scans<br>→ 20 children received sole IN dexmedetomidine<br><br><u>Median age:</u><br>3 years and 3 months<br><br><u>Median weight:</u><br>15,4 kg<br><br><u>**Inclusion criteria:**</u><br>- Retrospective audit: 28 February 2019 to 29 February 2020<br>- Prospective audit: 4 February to 15 October 2020<br>- Prospective study: 15 October 2020 to 21 May 2021<br><br><u>**Exclusion criteria:**</u><br>- Children who were not fasted<br>- Acutely unwell patients<br>- History of difficult airway<br>- Cardiac arrhythmia<br>- Neuromuscular disease<br>- Severe renal or hepatic impairment<br>- Using digoxin	<u>**Setting:**</u><br>Pediatric day hospital at North Middlesex Hospital<br><br><u>**Type of imaging:**</u><br>Magnetic resonance imaging (MRI scan)	<u>**Ramsay Sedation Score (RSS)**</u><br>→ *Used once after administration of chloral hydrate*<br>= score as a reference for additional administration of dexmedetomidine<br><br><u>**Monitoring**</u><br>Oxygen saturation:<br>- Continuous peripheral measurement<br>Heart rate and blood pressure:<br>- Baseline (presedation)<br>- Every 15-30 minutes after administration
**Purpose of the study**			**Method of administration, timing, dose**	
- To improve success rate of children requiring sedation for MRI.<br>- To assess the efficacy of three different protocols for pediatric sedation.			<u>**Method of administration:**</u><br>Dexmedetomidine 100mcg/ml was administered using a mucosal atomizer device<br><br><u>**NPO policy:**</u><br>- Min. 6 hours before for solids<br>- Min. 4 hours before for milk<br>- Min. 2 hours before for clear fluids<br><br><u>**Dose:**</u><br>- <15 kg: chloral hydrate + 2 µg/kg IN dexmedetomidine (if RSS <4)<br>- ≥15 kg or child who had failed sedation with another agent previously: 4 µg/kg IN dexmedetomidine	

Title, authors, date	Study design and characteristics	Sample, inclusion and exclusion criteria	Setting & type of imaging	Assessment tools
**Intranasal dexmedetomidine for paediatric sedation for diagnostic magnetic resonance imaging studies**<br><br>**Ambi et al. 2012** [21]<br><br>**Purpose of the study**<br><br>To determine whether 2 μg/kg intranasal dexmedetomidine offered effective sedation in children posted for diagnostic MRI studies.	**Design:**<br>Prospective, quasi-experimental study	**Sample:**<br>n = 28 children<br><br>Age:<br>Between 1 month and 10 years<br>Average weight:<br>10,7 kg<br>**Inclusion criteria:**<br>- Written informed consent from the parents/guardian<br>- Children aged up to 10 years of age<br>- Undergoing an MRI procedure<br>**Exclusion criteria:**<br>- General contraindications for MRI (i.e. cardiac pacemakers, neurostimulators, ferromagnetic implants etc).<br>- Known allergy to dexmedetomidine<br>- Presence of otorhynological diseases<br>- Children with major respiratory and cardiac diseases	**Type of imaging:**<br>Magnetic resonance imaging (MRI scan)<br><br>**Method of administration, timing, dose**<br><br>**Method of administration:**<br>All patients were administered with IN dexmedetomidine using tuberculin syringe<br><br>The bioavailability of nebulized sprays has been found to be superior to administration by drops into the nose.<br><br>**Timing:**<br>30 minutes before scheduled MRI scan<br><br>**Dose:**<br>2 μg/kg intranasal dexmedetomidine	**The University of Michigan Sedation Scale (UMSS)**<br>*The degree of sedation was assessed at 15 and 30 minutes*<br>→ A sedation score of ≥ 2 was considered satisfactory<br>**Five grade scale:** *MRI image quality*<br>- Grade 0 or 1: the examination was of no or very little diagnostic usefulness because of extensive motion artifacts<br>- Grade 2: allowed to make the diagnosis, but some motion artifacts were still present<br>- Grade 3 and 4: good or excellent image quality, with no or almost absent motion artifacts<br>**Modified Aldrete score**<br>→ *Five criteria*<br>**- Activity:**<br>2 = Able to move 4 extremities voluntarily or on command<br>1 = Able to move 2 extremities voluntarily or on command<br>0 = Unable to move extremities voluntarily or on command<br>**- Respiration:**<br>2 = Able to breathe deeply and cough freely<br>1 = Dyspnea or limited breathing<br>0 = Apneic<br>**- Circulation:**<br>2 = Blood pressure +/- 20% of pre-anesthetic level<br>1 = Blood pressure +/- 20-49% of pre-anesthetic level<br>0 = Blood pressure +/- 50% of pre-anesthetic level<br>**- Consciousness:**<br>2 = Fully awake<br>1 = Arousable on calling<br>0 = Not responding<br>**- Oxygen saturation:**<br>2 = Able to maintain O_2 saturation >92% on room air<br>1 = Needs O_2 inhalation to maintain O_2 saturation >90%<br>0 = O_2 saturation <90%, even with O_2 supplement

Title, authors, date	Study design and characteristics	Sample, inclusion and exclusion criteria	Setting & type of imaging	Assessment tools
**Intranasal Dexmedetomidine for Sedation for Pediatric Computed Tomography Imaging.**<br><br>**Filho et al. 2015** [22]	**<u>Design:</u>**<br>Prospective, observational pilot study<br><br>**<u>Blinding:</u>**<br>Two radiologists were blinded to the sedation technique	**<u>Sample:</u>**<br>n = 60<br>→ 63 CT studies<br><br><u>Average age:</u><br>17,1 months<br><br><u>Average weight:</u><br>10,7 kg<br><br>**<u>Inclusion criteria:</u>**<br>- Children in the ED in need of a CT imaging study<br>- 1 month to 5 years of age<br>- ASA status I /II<br>- Informed parental consent<br>- No need for or presence of an IV catheter<br>- No clinical evidence of vomiting, reflux or aspiration<br>- No contraindications for dexmedetomidine therapy<br><br>**<u>Exclusion criteria:</u>**<br>- (Need for) IV catheter<br>- Evidence of vomiting, reflux, or aspiration.<br>- Contraindications to dexmedetomidine therapy.	**<u>Type of imaging:</u>**<br>Computed tomography (CT scan)	**<u>Monitoring:</u>** *physiological measurements*<br>- Baseline<br>- Every 5 minutes after dexmedetomidine administration<br><br>**<u>Modified Aldrete score</u>**<br>Minimum score of 9 = discharge<br><br>**<u>Ramsay Sedation Scale (RSS)</u>**<br>*After 15 minutes the depth of sedation was assessed*<br>→ A minimum RSS score of 3 was required to ensure motionless conditions<br>→ RSS score < 3: a second dose of 1 $\mu g.kg^{-1}$ IN dexmedetomidine was administered<br><br>**<u>World Society of Intravenous Anesthesia Sedation Outcome Tool</u>**<br>- Step 1: was there one or more adverse events associated with this sedation encounter?<br>- Step 2: describe the adverse events.<br>- Step 3: note the interventions performed to treat the adverse events.<br>- Step 4: note the outcome of the adverse events.<br>- Step 5: assign a severity rating to the adverse events associated with this sedation event.
**Purpose of the study**			**Method of administration, timing, dose**	
Evaluation of the aerosolized intranasal route for dexmedetomidine as a safe, effective, and efficient option for infant and pediatric sedation for CT.			**<u>Method of administration:</u>**<br>- Administration with a nasal mucosal atomizer device (Wolfe Tory Medical) by an emergency medicine nurse, under the direct supervision of a pediatrician.<br>- For optimal delivery, the child was positioned either supine or sitting with the head tilted back.<br><br>**<u>NPO policy:</u>**<br>→ *ACEP guidelines*<br>= recent food intake is not a contraindication for administering procedural sedation and analgesia, but should be considered in choosing the timing and target level of sedation<br><br>**<u>Dose:</u>**<br>2.5 µg/kg intranasal dexmedetomidine	

Title, authors, date	Study design and characteristics	Sample, inclusion and exclusion criteria	Setting & type of imaging	Assessment tools
**Pharmacokinetics and Sedative Effects of Intranasal Dexmedetomidine in Ambulatory Pediatric Patients**<br><br>**Uusalo et al. 2020** [23]	**<u>Design:</u>**<br>Open-label, exploratory study without randomization	**<u>Sample</u>:** n = 50<br>**<u>Inclusion criteria:</u>**<br>1 month to 11 years of age; Written informed consent from the patients' legal guardians + assent > 6 years of age; Guardians fluent in Finnish/Swedish; Scheduled to receive dexmedetomidine for sedation as part of their clinical care during MRI<br>**<u>Exclusion criteria:</u>**<br>Newborns; History of intolerance to the study drug or to related compounds; Previous drug therapy with dexmedetomidine in the 14 days before the study; Use of stimulants or any drugs known to cause enzyme induction; Existing or recent disease that could influence the study outcome or cause a health hazard; Clinically abnormal findings in physical examination or laboratory screening; Patients participating in any other clinical study involving drug products concomitantly or within 1 month before the entry into this study	**<u>Setting:</u>**<br>Turku University Hospital in Finland<br><br>**<u>Type of imaging:</u>**<br>Magnetic resonance imaging (MRI scan)	**<u>Comfort-B Sedation Scale (CBSS)</u>**<br>*To assess the psychomotor effects of IN dexmedetomidine*<br>- Alertness<br>- Calmness/agitation<br>- Respiratory response<br>- Physical movement<br>- Muscle tone<br>- Facial tension<br>= 6-10: oversedation<br>= 11-23: moderately sedated<br>= 24-30: little sedated - insufficiently sedated!<br>→ Sedation was assessed clinically acceptable if CBSS decreased ≥ 6 points<br>**<u>Continuous monitoring</u>**<br>*After drug administration*<br>- Heart rate<br>- Peripheral oxygen saturation<br>**<u>Visual inspection</u>**<br>*Local tolerability of IN dexmedetomidine was assessed and recorded real time (crying, nasal irritation and runny nose)*<br>- Immediately during administration<br>- After 1 hour<br>- After 2 hours<br>- After 3 hours<br>- After 4 hours<br>- At the end of the clinical observation period
**Purpose of the study**			**Method of administration, timing, dose**	
To evaluate the absorption and pharmacokinetics of IN dexmedetomidine after 2-3 μg.kg^{-1} dose in pediatric patients scheduled for MRI requiring sedation.<br><br>To compare the pharmacological effects caused by sole IN dexmedetomidine to pharmacokinetics during pediatric sedation.			**<u>Method of administration:</u>**<br>Use of a nebulizer (LMA® MAD NasalTM)<br>**<u>Timing:</u>**<br>+/- 45-60 minutes before the scheduled MRI procedure<br>**<u>Dose:</u>**<br>*Actual dose determined by the anesthesiologist taking care of the patient*<br>→ 0 - 2 years: average 2,7 μg/kg IN dexmedetomidine<br>→ 2 - 6 years: average 2,9 μg/kg IN dexmedetomidine	

Title, authors, date	Study design and characteristics	Sample, inclusion and exclusion criteria	Setting & type of imaging	Assessment tools
**The Use of Intranasal Dexmedetomidine and Midazolam for Sedated Magnetic Resonance Imaging in Children**<br><br>**Sulton et al. 2017** [24]	**<u>Design:</u>**<br>Prospective, observational study<br><br>**<u>Blinding:</u>**<br>The dataset was blinded to the institution	**<u>Sample:</u>**<br>n = 256<br><br><u>Average age:</u><br>14 months<br><br>**<u>Inclusion criteria:</u>**<br>- Sedations performed by anesthesiologists, pediatric physicians, nurses, dentists, physician assistants, and other health care personnel<br>- Data were collected on patients between 2007 and 2011<br><br>**<u>Exclusion criteria:</u>**<br>- Patients who received sedative medication via the intravenous route<br>- Patients who received chloral hydrate	**<u>Setting:</u>**<br>Pediatric Sedation Research Consortium (PSRC)<br>→ 42 institutions that are committed to gathering data prospectively on all pediatric sedations at their respective institutions<br><br>**<u>Type of imaging:</u>**<br>Magnetic resonance imaging (MRI scan)	**<u>Previous reports from the PSRC</u>**<br>*Used as a reference:*<br>→ <u>Major adverse events:</u><br>Aspiration, death, cardiac arrest, unplanned hospital admission or level-of-care increase, or emergency anesthesia consultation.<br><br>→ <u>Minor adverse events:</u><br>Apnea, airway obstruction, desaturation, bradycardia, hypotension, and inability to complete the procedure.
**Purpose of the study**			**Dose**	
To describe the use of intranasal dexmedetomidine (in combination with midazolam) for sedated magnetic resonance imaging examinations in children.			**<u>Median dose:</u>**<br>3 μg/kg intranasal dexmedetomidine	

Study	Outcomes and conclusions	Side effects (+ potential interventions)
**Yuen et al. 2017 [19]**	**<u>Primary outcomes:</u>**<br>- Difference in time to recovery:<br>= median time of 4,3 hours after IN dexmedetomidine<br>= median time of 5 hours after PO chloral hydrate<br>} *= no significant difference (p = 0,36)*<br>- Resumption of normal activities 4 hours after discharge:<br>= 42% recovered after 4 hours (IN dexmedetomidine)<br>= 39% recovered after 4 hours (PO chloral hydrate)<br>} *= no significant difference (p = 0.76)*<br>- Mean time to sedation after IN dexmedetomidine:<br>= 19,6 minutes<br>→ 2,8 minutes faster than chloral hydrate<br>} *= significant difference (p = 0.03)*<br>**<u>Secondary outcomes:</u>**<br>- Successful sedation (n=64)<br>- Respiratory event (n=0)<br>- More children cried or resisted after drinking chloral hydrate syrup compared to placebo syrup<br>→ 72/107 (67%) vs. 42/87 (48%) = *significant difference (p = 0.009)*<br>→ Chloral hydrate can cause nausea or vomiting + children disliked the bitter taste<br>**<u>Conclusions</u>: The successful sedation of children before CT studies is similar after oral chloral hydrate or intranasal dexmedetomidine (3 µg/kg). Dexmedetomidine is associated with better behavior and less gastrointestinal side effects. IN dexmedetomidine appears to be a safer and more acceptable method of sedating children.**	- Hypotension (n=9)<br>- Bradycardia (n=14)<br>**<u>First 24 hours at home:</u>**<br>Registered by parents<br>- Sleepy<br>- Unsteady (n=0)<br>- Hyperactive<br>- Anorexic<br>- Vomiting (n=0)
**Ghai et al. 2016 [17]**	**<u>Primary outcomes:</u>**<br>- Incidence of children having RSS ≥ 4 on the CT table (not requiring IV sedation):<br>= 67% (dexmedetomidine)<br>= 24% (midazolam)<br>} *= significant difference (p = 0.002)*<br>**<u>Secondary outcomes:</u>**<br>- Parental satisfaction<br>→ dexmedetomidine group: majority of the parents was 'satisfied' (n=24)<br>- Number of venipuncture attempts *= no significant difference (p = 0.05)*<br>- Movements and motion artifacts during scan (n=1) *= no significant difference (p = 0.53)*<br>- Requirement of repeat scan (n=0)<br>- GDRS	- Oxygen desaturation (n=0)<br>- Airway obstruction (n=0)<br>- Bradycardia (n=0)<br>- Vomiting (n=0)<br>→ None of the children vomited or spat out the drug.<br>→ Following administration of premedication, oxygen saturation was monitored.

	→ lower median scores were noted in dexmedetomidine group at the time of venipuncture<br>*= significant difference (p = 0.04)*<br>- Mean requirement of IV ketamine for CT imaging<br>= 33% (dexmedetomidine)<br>= 6% (midazolam)<br>- Peak plasma concentrations<br>= after 38 minutes (dexmedetomidine)<br>= after 31 minutes (midazolam)<br>- Mean discharge time<br>= 39,5 minutes<br>→ 93,33% had good acceptance to the premedication administered<br>**<u>Conclusions</u>: Intranasal dexmedetomidine is superior to oral midazolam in producing satisfactory sedation in greater number of children for carrying out CT imaging. Dexmedetomidine premedication may provide clinical benefits including reduction in requirements of additional intravenous sedatives**.	→ All children were observed after the procedure till discharge criteria were met.
**Tug et al. 2015 [18]**	**<u>Primary outcomes:</u>**<br>- Onset time of sedation:<br>= 31 minutes (group 1)<br>= 30 minutes (group 2)<br>*= no significant difference (p = 0.570)*<br>**<u>Secondary outcomes</u>**:<br>- Parental separation score:<br>= 1 : group 1: n=13; group 2: n=7<br>= 2 : group 1: n=10; group 2: n=3<br>= 3 : group 1: n= 7 ; group 2: n=20<br>*= significantly higher in group 2 (p = 0.003)*<br>- RSS*:* varies in both groups<br>- BIS *significant difference in groups prior to MRI scan (p = 0.000), higher score in group 1*<br>- Heart rate *no significant differences*<br>- Number of patients requiring rescue medication:<br>= 70% (group 1)<br>= 30% (group 2)<br>*= significant difference (p = 0,002)*<br>- Sedation duration:<br>= 72 minutes (group 1)<br>= 65 minutes (group 2)<br>*= no significant difference (p = 0,249)*<br>- Parents' satisfaction	- Respiratory depression (RR <12/min)<br>- Desaturation (n=0)<br>- Bradycardia (HR <70 beats/min)<br>- Allergic reactions<br>→ No adverse effects were observed during the sedation procedure.<br>**<u>Interventions:</u>**<br>If SpO_2, RR and HR fell below the expected levels, oxygen via face mask was given and/or intravenous atropine was injected.

	= 1 : group 1: n=2; group 2: n=0<br>= 2 : group 1: n=13; group 2: n=9<br>= 3 : group 1: n= 15 ; group 2: n=21 } = *no significant difference (p = 0,114)*<br>- Recovery duration:<br>= 56 minutes (group 1)<br>= 46 minutes (group 2) } = *no significant difference (p = 0,057)*<br><br>All MRI examinations were completed successfully, and there was no requirement for additional scans.<br>__Conclusions__: **4 µg/kg intranasal dexmedetomidine was found to be superior to 3 µg/kg intranasal dexmedetomidine without affecting hemodynamics and respiration (= efficient and safe agent).**<br>→ __**Benefits of 4 µg/kg:**__<br>- **Reduced requirement of rescue drugs**<br>- **Better parental separation mood**<br>- **Lower BIS values**<br>- **Higher sedation scores**	
**Jackson et al. 2021 [20]**	__**Primary outcomes:**__<br>- Success rate:<br>= 76,2% for scans using 4 µg/kg intranasal dexmedetomidine<br>= 33,3% for scans using midazolam } = *significant difference (p < 0.05)*<br>__Conclusions__: **Intranasal dexmedetomidine is the most effective agent. Intranasal dexmedetomidine is effective as an alternative to oral midazolam and as a rescue medication after failed chloral hydrate. Dexmedetomidine is the most successful agent in patients who had a failed sedation earlier. The hemodynamic changes associated with dexmedetomidine were similar to those reported in other larger series, none requiring intervention.**	(Observations of 18/21 children)<br>- 13/18 had a HR lower than the advanced pediatric life support (APLS) normal range.<br>- 2/18 had a systolic blood pressure below APLS normal range.<br>No patient required any interventions after review by a clinician, no adverse events noted.
**Ambi et al. 2012 [21]**	__**Primary outcomes:**__<br>- Mean sedation scores (UMSS) after 15 minutes = 1,17<br>- Mean sedation scores (UMSS) after 30 minutes = 2,60<br>- Mean discharge time (according to modified Aldrete score) = 81,39 minutes<br>__**Secondary outcomes:**__<br>- Success rate of 60% (no need for IV midazolam)<br>- 11/28 were deemed as failed cases and had to be supplemented with IV midazolam (= 40%)<br>→ All the children accepted parental separation well.	

	<u>Conclusions</u>: **Dexmedetomidine may be a useful agent for sedation of children undergoing MRI studies. Use of a meter-dozed atomizer device or concurrent use of benzodiazepines may enhance the success rate.**	
**Filho et al. 2015 [22]**	**<u>Primary outcomes:</u>**<br>- Average time to achieve sedation: 13,4 minutes<br>- Time to meet discharge criteria (minimum modified Aldrete Score 9) : 88,7 minutes<br>- All patients were successfully sedated.<br>→ 3 patients (5%) required a second dose of 1 µg/kg<br>**<u>Secondary outcomes:</u>**<br>- In 100% of scans, image quality was graded as "excellent" and without motion or image artifacts.<br>- Average NPO time = 206.3 minutes<br>**<u>Conclusions:</u>**<br>**Equally safe, but more effective as aerosolized midazolam; less need for a second dose compared with intranasal midazolam; better image quality; better success for sedation. Intranasal dexmedetomidine can produce successful, high-quality CT imaging conditions within 13 minutes of administration, with discharge to home within 90 minutes of the initial dose.**	- Prolonged recovery time (n=1)<br>- Hypoxia (n=1)<br>- Vomiting (n=1)<br>- >20% decrease in HR (n=9)<br>- >20% drop in mean arterial blood pressure (n=1)<br>- 20% increase in mean arterial blood pressure (n=2)<br>- 20% increase in heart rate (n=1)<br>No pharmacological interventions needed related to hemodynamic changes.
**Uusalo et al. 2020 [23]**	**<u>Primary outcomes:</u>**<br>Peak plasma concentration (C_{max}) and time to achieve C_{max} (T_{max}) after IN dexmedetomidine administration<br>- C_{max} (0 - 2 years): 0,46 ng.ml<br>- C_{max} (2 - 6 years): 0,51 ng.ml<br>- T_{max} (0 - 2 years): 35 min<br>- T_{max} (2 - 6 years): 32 min<br>The concentration–time profiles indicated that the plasma concentrations decreased quite rapidly<br>→ higher initial doses or repeated dosing may be needed for clinical efficacy for longer procedures.<br>**<u>Secondary outcomes:</u>**<br>- CBSS:<br>→ maximal decline from baseline was 8 points after dexmedetomidine<br>→ *significant difference, negatively correlated to* C_{max} *($p < 0.016$)*<br>→ maximal reduction was recorded 45 minutes after dosing<br>- Administration of thiopental:<br>→ n = 47<br>→ Because most of the patients received additional sedation during MRI, they were not able to evaluate the duration of sedation caused by dexmedetomidine alone.<br>- Intranasal dexmedetomidine tolerability:	- Maximal decrease in HR:<br>o 0 – 2 years: median 20 beats/min (15% relative change)<br>o 2 – 6 years: median 17 beats/min (15% relative change)<br>→ intranasal dexmedetomidine reduced HR less compared to IV dosing, which produces up to 30% decreases in HR.<br>→ Similar to previous reports on IN dexmedetomidine, HR was reduced most in the youngest patients of 0–2 years of age. The risk of bradycardia should be kept in mind when using high dexmedetomidine doses in young patients.

	o Crying (n=16)<br>o Runny nose (n=8)<br>o Nasal irritation (n=0)<br>o Vomiting (n=1)<br>→ They were not able to measure the absolute bioavailability in this single-period observational study, but the results show that nasally applied dexmedetomidine is efficiently absorbed in children.<br><u>Conclusions</u>: **Intranasal dexmedetomidine is relatively rapidly absorbed and causes significant sedation in pediatric patients. Pharmacokinetics of IN dexmedetomidine in pediatric patients quite similar compared to adults. The results suggest that IN dexmedetomidine as sole agent might not be sufficient for procedural MRI sedation of pediatric patients. Combination with other sedative agents may be needed.**	- No differences in saturation<br>- Vomiting (n=1)<br>- No serious adverse events related to study drug were reported. The reported adverse effects were mild and mostly related to an unpleasant feeling in nasal mucosa immediately after administration of the nasal spray.
**Sulton et al. 2017 [24]**	**<u>Primary outcomes:</u>**<br>- Major adverse events:<br>→ no complications recorded<br>- Minor advers events:<br>→ no complications recorded<br>→ No intervention was required in 197/224 of the patients .<br>→ For patients that did require intervention, blow by mask oxygen was the most common followed by repositioning.<br>→ One patient required placement of an oropharyngeal airway.<br>→ Interventions for cardiovascular changes are uncommon.<br><u>Conclusions</u>: **A notable difference in this report is the addition of midazolam as an adjunct medication. A possible explanation may relate to the difference in stimulation involved in obtaining a CT examination, auditory brainstem response examination, or transesophageal echocardiography as compared with an MRI examination. They speculate that the increased auditory stimulation of an MRI examination requires a deeper level of sedation. This is consistent with other reports noting higher sedative dose to accomplish MRI examinations as compared with CT examinations. Intranasal dexmedetomidine combined with midazolam can be an effective sedation agent for pediatric MRI**.	/
